# Difficult Cases

**Published:** 1888-07-21

**Authors:** S. Bulkeley Jacson

**Affiliations:** 19, Curzon Park, Chester.


### DIFFICULT CASES.

[Replies endorsed "Difficult Cases," should be addressed to the Editor.]

I hope you will kindly pardon my troubling you, but I have been recommended to apply to you for help on behalf of William Gresty, a youth aged nineteen, for whom I am anxious to find some employment. Several people who have employed him say that he is most strictly honest and trustworthy. He is able to clean boots and knives, and to run errands; but, being hunchbacked, he is not fitted for a house-servant's place. The poor lad is very anxious to obtain a situation, as his brother, with whom he now lives, is unable to keep him any longer. I should be so grateful if any readers of your paper would try the boy, as I feel quite sure he would do well, with kind treatment. His brother does not wish him to go very far away from Chester. The clergyman of the parish will speak most highly of him, and I should also be able to get another reference for him from a lady who has known him for some time and employed him in various ways. If you are unable to help me, could you kindly suggest some other way of obtaining a situation for the lad?

S. Bulkeley Jackson.

19, Curzon Park, Chester.

[We should be glad if any of our readers would give a helping hand to this poor youth, or put him in the way of getting suitable employment.—Ed. *T. H.*]

